# Supplementary material for: Dynalign II: common secondary structure prediction for RNA homologs with domain insertions
Source: Nucleic Acids Res. 2014 Nov 21;42(22):13939–48. doi: 10.1093/nar/gku1172 (PMC4267632; doi:10.1093/nar/gku1172)
Supplement: SUPPLEMENTARY DATA [file supp_gku1172_nar-02021-z-2014-File012.zip › manual/javadoc/RNAstructure_java_drawing/source/runners/package-frame.html]

RNAstructure\_java\_drawing.source.runners


# RNAstructure\_java\_drawing.source.runners

## Classes

- PlotRunner
- RunnerTemplate
- StructureRunner
